# Potential Pitfall in the Assessment of Lung Cancer with FDG-PET/CT: Talc Pleurodesis Causes Intrathoracic Nodal FDG Avidity

**DOI:** 10.1155/2013/683582

**Published:** 2013-05-25

**Authors:** Yingbing Wang, Brett W. Carter, Victorine Muse, Subba Digumarthy, Jo-Anne Shepard, Amita Sharma

**Affiliations:** Department of Radiology, Division of Thoracic Imaging, Massachusetts General Hospital, 55 Fruit Street, Boston, MA 02114, USA

## Abstract

*Objective*. Talc pleurodesis is a common procedure performed to treat complications related to lung cancer. The purpose of our study was to characterize any thoracic nodal findings on FDG PET/CT associated with prior talc pleurodesis. *Materials and Methods*. The electronic medical record identified 44 patients who underwent PET/CT between January 2006 and December 2010 and had a history of talc pleurodesis. For each exam, we evaluated the distribution pattern, size, and attenuation of intrathoracic lymph nodes and the associated standardized uptake value. *Results*. High-attenuation intrathoracic lymph nodes were noted in 11 patients (25%), and all had corresponding increased FDG uptake (range 2–9 mm). Involved nodal groups were anterior peridiaphragmatic (100%), paracardiac (45%), internal mammary (25%), and peri-IVC (18%) nodal stations. Seven of the 11 patients (63%) had involvement of multiple lymph nodal groups. Mean longitudinal PET/CT and standalone CT followups of 15 ± 11 months showed persistence of both high-attenuation and increased uptake at these sites, without increase in nodal size suggesting metastatic disease involvement. *Conclusions*. FDG avid, high-attenuation lymph nodes along the lymphatic drainage pathway for parietal pleura are a relatively common finding following talc pleurodesis and should not be mistaken for nodal metastases during the evaluation of patients with history of lung cancer.

## 1. Introduction

Since pleurodesis was first introduced in the early 19th century, it has proven to be an effective therapy for preventing recurrent pleural effusions and pneumothoraces. By far, talc pleurodesis is the most widely used method for obliterating the pleural space and is frequently performed for the palliation of lung cancer. The sequelae of talc pleurodesis in the pleura has been described in the literature and can be detected on 18F-2-fluoro-2-deoxyglucose (FDG) PET/CT imaging both by areas of high-attenuation pleural thickening and increased FDG uptake that persists over serial exams [1–5]. Once administered, talc is not confined to the pleural space. In rabbit models, talc has been shown to be absorbed through the parietal pleura into mediastinal lymph nodes and the thoracic duct, eventually entering the systemic lymphatic circulation [6]. To our knowledge, no studies have characterized the extra pleural sequelae of talc pleurodesis in humans, and more specifically, the pattern of lymph node involvement. We postulated that we would find similar features of high-attenuation and increased uptake in nodes, as found in pleura. As in pleura, talc deposition in intrathoracic nodal groups should incite a granulomatous reaction, resulting in increased FDG uptake that can potentially mimic tumor and would be an important pitfall to recognize. Therefore, the aim of our study was to identify and characterize the imaging features associated with prior talc deposition in intrathoracic lymph nodes. 

## 2. Subjects and Methods

Institutional Review Board approval was obtained for this retrospective study, and informed consent was waived. 

### 2.1. Patient Identification

Our institution's radiology exam database was queried in the period between January 2006 and December 2010 for all FDG PET/CT exams with pleural findings characteristic of prior talc pleurodesis. Our electronic medical records were queried to confirm a prior history of talc pleurodesis for each patient. Additional relevant clinical information was also obtained, including the cancer histology, cancer stage at diagnosis, treatment history, biopsy results, and indications for talc pleurodesis and for FDG PET/CT. 

### 2.2. Integrated FDG PET/CT Scan

For each PET/CT study, the patient underwent a low-dose nondiagnostic CT for attenuation correction purposes only, a separate diagnostic CT with or without iodinated contrast, and an FDG PET scan. Separate low-dose attenuation correction and diagnostic CTs were performed to avoid errors in attenuation correction related to intravenous and oral contrast. The resulting PET and CT images were fused, and the images were viewed in the axial, coronal, and sagittal planes on a dedicated workstation using Syngo TrueD software (Siemens Medical Solutions, Erlangen, Germany). 

The nondiagnostic, low-dose attenuation correction CT was performed using 120 kVp, modulated mA dependent on patient BMI (11 mA for BMI less than 30, 30 mA for BMI between 30 and 34, 40 mA for BMI between 35 and 44, and 100 mA for BMI exceeding 45), a tube rotation time of 0.5 seconds per rotation, a pitch of 1.5, and a section thickness of 5 mm. These were low-dose scans with a maximal mA of 100 in the heaviest patients. A separate diagnostic CT with or without iodinated intravenous contrast was obtained using 120 kVp, modulated mA, a tube rotation time of 0.5 seconds per rotation, a variable pitch between 1 and 1.5, depending on patient BMI, and a section thickness of 2.5 mm. For the CT scans, the patients were instructed to hold their breath at mid inspiration. Immediately after the CT scan was obtained, a PET scan was obtained over the same anatomical regions in 7 table positions, at 3 minutes per bed position for patients with BMI less than 30, 4 minutes per bed position for BMI between 30 and 34, 5 minutes per bed position for BMI between 35 and 44, and 6 minutes per bed position for BMI exceeding 45. PET data was reconstructed iteratively with segmented correction for attenuation with the CT data. Final section thickness was 5 mm. Each patient fasted for a minimum of 6 hours prior to administration of FDG. A blood glucose level was obtained immediately prior to the study to exclude hyperglycemic patients. The mean glucose level was 110 ± 15 mg/dL in our study cohort. A single intravenous injection of 15.8 ± 2.2 mCi (584 ± 81 MBq) of FDG was administered if the glucose level was <250 mg/dL. The patients were scanned approximately 45 minutes after injection in an integrated PET/CT scanner (Siemens Medical Systems, Malvern, PA, USA) consisting of a 64-slice PET/CT. The patients were instructed not to hold their breath but to breathe shallowly during the PET scan. 

### 2.3. Image Analysis

Two board certified radiologists with 1–8 years of experience in interpreting PET/CTs independently reviewed all FDG PET/CT exams available for each patient in the study. On CT, each radiologist recorded the location, size, and attenuation of lymph nodes that appeared high-attenuation by visual inspection. We only defined areas of high-attenuation as nodes if they were focal, displayed convex borders, and separate from more curvilinear areas of high-attenuation in pleura. To distinguish anterior peridiaphragmatic lymph nodes from pleural thickening along the costophrenic sulci, multiplanar imaging was used. Attenuation was always measured on the nondiagnostic, low-dose CT, performed without IV contrast, to avoid misinterpretation of enhancement as intrinsic high-attenuation in lymph nodes. For attenuation and SUV measurements, we drew an elliptical ROI within the margins of the finding. We used a histogram box with manual adjustment of size to fit the node of interest in order to measure the maximum attenuation. 

Only lymph nodes that measured greater than 100 HU were considered to be involved by talc dissemination. Any available chest CTs performed prior to talc pleurodesis were also reviewed to exclude preexisting high-attenuation nodes related to nontalc-related calcification or granuloma formation. The attenuation of high-attenuation pleural thickening was also measured.

On PET, the radiologists determined the degree of uptake for each high-attenuation lymph node by subjective visual inspection (“hot” or “not” relative to mediastinal background) and objective quantitative measurement of the mean and maximum SUV. Both the attenuation-corrected and non attenuation-corrected FDG PET images were reviewed in order to exclude artifactually increased uptake related to attenuation correction of high-attenuation tissue. Uptake in high-attenuation pleural thickening was also recorded. In instances where the two readers disagreed on any findings, a third adjudication reader with board certification in diagnostic radiology and 8-year experience in interpreting PET/CTs evaluated the study, and this interpretation was used in the results. 

### 2.4. Statistics

A paired *t*-test was used to determine if the measured mean SUV for anterior peridiaphragmatic and peri-IVC lymph nodes was significantly different from the combined mean SUV of lymph nodes far from the diaphragm. This was performed to assess whether respiration significantly affected the measured FDG avidity of lymph nodes near the diaphragm. The same test was used to determine if densities of high-attenuation lymph nodes were significantly different compared to areas of high-attenuation pleural thickening. 

A chi-square test was applied to determine if sex, indication for pleurodesis, lung cancer histology, or stage of disease differed significantly between groups with or without FDG avid high-attenuation lymph nodes. 

## 3. Results

### 3.1. Patients

Forty-four patients were identified with a confirmed history of talc pleurodesis and reported pleural imaging findings on FDG-PET/CT characteristic for talc pleurodesis. Mean patient age was 64 ± 12 years, with a range between 34 and 81. Twenty-one patients (47%) were males, and twenty-three patients were females (53%). The indications for talc pleurodesis included 17 cases of malignant effusion (39%), 11 cases of persistent air leak following thoracotomy (25%), 5 cases of pneumothorax (11%), and 11 cases of recurrent nonmalignant pleural effusions (25%), including 2 cases of chylothorax. Patient demographics are summarized in Table 1. 

Forty-one patients had known lung cancer and their FDG PET/CT scans were performed for purposes of staging, restaging, or surveillance. In the three remaining patients, the indications included assessment of treatment response in pulmonary sarcoidosis in one and evaluation for occult malignancy in two other patients. 

Mean time interval between talc pleurodesis and PET/CT was 32 ± 37 months. The distribution was non-Gaussian and skewed toward longer intervals, with 75% of patients undergoing PET/CT within 30 month of their pleurodesis and 25% of patients undergoing PET/CT imaging within 7 months of pleurodesis; however, six patients underwent PET/CT within 10 days of their talc pleurodesis procedure. 

### 3.2. High-Attenuation Lymph Nodes

High-attenuation lymph nodes were observed on noncontrast CT in 11 of 44 patients (25%). The mean density of high-attenuation lymph nodes was 130 HU ± 29. A preprocedural chest CT was available for 8 patients with FDG avid and high-attenuation lymph nodes (72%). None of these chest CTs demonstrated high-attenuation lymph nodes prior to talc pleurodesis. 

**Figure 1:**
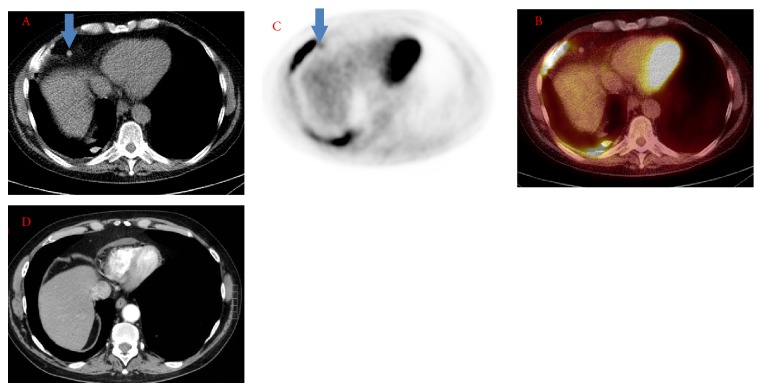
PET/CT images showing CT (A), PET (B), and fused images (C). There is an FDG avid and high-attenuation peridiaphragmatic lymph node (arrow) adjacent to a curvilinear area of FDG avid and high-attenuation pleural thickening. There is slight misregistration related to lateral excursion of the chest during respiration. A chest CT (D) performed prior to talc pleurodesis does not show any preexisting high-attenuation peridiaphragmatic lymph node.

**Figure 2:**
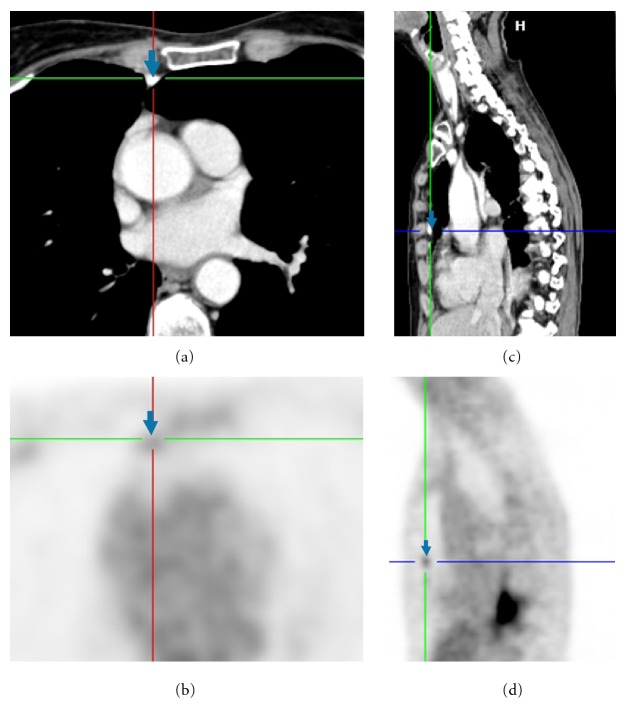
A Patient with avid and high-attenuation right internal mammary lymph node (arrow) on axial CT (a), axial PET (b), sagittal CT (c), and sagittal PET (d) images. Chest CT images prior to talc pleurodesis are not available.

All high-attenuation lymph nodes were associated with increased FDG uptake by visual and quantitative assessment (Figures 1 and 2). The anterior peridiaphragmatic nodes were affected in all 11 patients (100%). 5 patients had paracardiac (45%), 4 patients had internal mammary (25%), and 2 patients had peri-IVC (18%) high-attenuation nodal groups. Seven of the 11 patients (63%) had involvement of multiple lymph nodal groups. Maximum and median SUV uptake was 2.5 ± 1.3 and 2.3 ± 1.1, respectively, for high-attenuation lymph nodes. The average lymph node size was 5 ± 1 mm, with a range of 2 to 9 mm. The size, attenuation, and FDG uptake data for lymph nodes, stratified by nodal station, are summarized in Table 2. The demographics of patients with high-attenuation FDG avid nodes compared with patients who did not are summarized in Table 1. 

### 3.3. Pleura

All patients showed FDG avid high-attenuation pleural thickening on the side of prior treatment. Overall, maximum and median SUV uptake were 6.2 ± 3.1 and 5.1 ± 2.7, respectively, for high-attenuation pleural thickening. The mean density of high-attenuation pleural thickening was 160 HU ± 80. 

### 3.4. Followup

Clinical and imaging followup for patients ranged from 1 to 54 months, with an average of 15 ± 11 months. Thirty-seven patients (84%), including all patients with high-attenuation lymph nodes, had CT chest followup demonstrating stability in size of high-attenuation lymph nodes without evidence for progressive disease at these sites. Increased uptake associated with high-attenuation nodes also persisted on longitudinal imaging. 

### 3.5. Statistics

There was no significant statistical difference between mean FDG uptake in anterior peridiaphragmatic and peri-IVC nodal uptake (SUV, 2.3 ± 1.1) compared to combined mean FDG uptake of other high-attenuation nodal groups far from the diaphragm (SUV, 2.4 ± 1.2), with *P* = 0.83. There was no statistical significance between density of high-attenuation lymph nodes (130 HU ± 29) and high-attenuation pleural thickening (160 HU ± 80) with *P* = 0.31. 

The proportion of patients of male sex, malignant indication for pleurodesis, NSCLC histology, or advanced stage of disease did not differ between patients with FDG avid high-attenuation lymph nodes from those who did not (*P* > 0.05). 

## 4. Discussion 

While the sequelae of talc pleurodesis in pleura has been described in the literature, there has been a shortage of discussion of other intrathoracic findings. In our study, we observed that high-attenuation intrathoracic lymph nodes are common following talc pleurodesis and occur in the anterior peridiaphragmatic, paracardiac, internal mammary, and peri-IVC nodal groups, which are part of the lymphatic pathway for parietal pleural drainage [7]. This finding is consistent with previously published work on the distribution pattern for talc in rabbit models and compatible with the route of talc dissemination [6]. We did not observe any high-attenuation lymph nodes in ipsilateralhilar or periesophageal regions, which comprise the lymphatic pathway for visceral pleural drainage. A preprocedural chest CT was available for 72% (8/11) patients with FDG avid and high-attenuation lymph nodes. None of these chest CTs demonstrated high-attenuation lymph nodes prior to talc pleurodesis, excluding the possibility that these lymph nodes represented calcified granulomas (Figure 1). 

Overall, the FDG uptake in high-attenuation lymph nodes (2.5 ± 1.3) was less than that for high-attenuation pleural thickening (5.1 ± 2.7). This is likely related to the small size of lymph nodes, which were all subcentimeter and ranged between 2 and 9 mm. The average size was 5 mm, which is at the limits of detection by PET. These small lymph nodes demonstrate quantitatively significant and visually apparent uptake, suggesting substantial underlying FDG avidity, as would be expected from talc induced inflammation. Increased FDG uptake was observed on both attenuation-corrected and nonattenuation-corrected PET images, excluding false positive uptake related to attenuation correction of high-attenuation tissue. 

**Figure 3:**
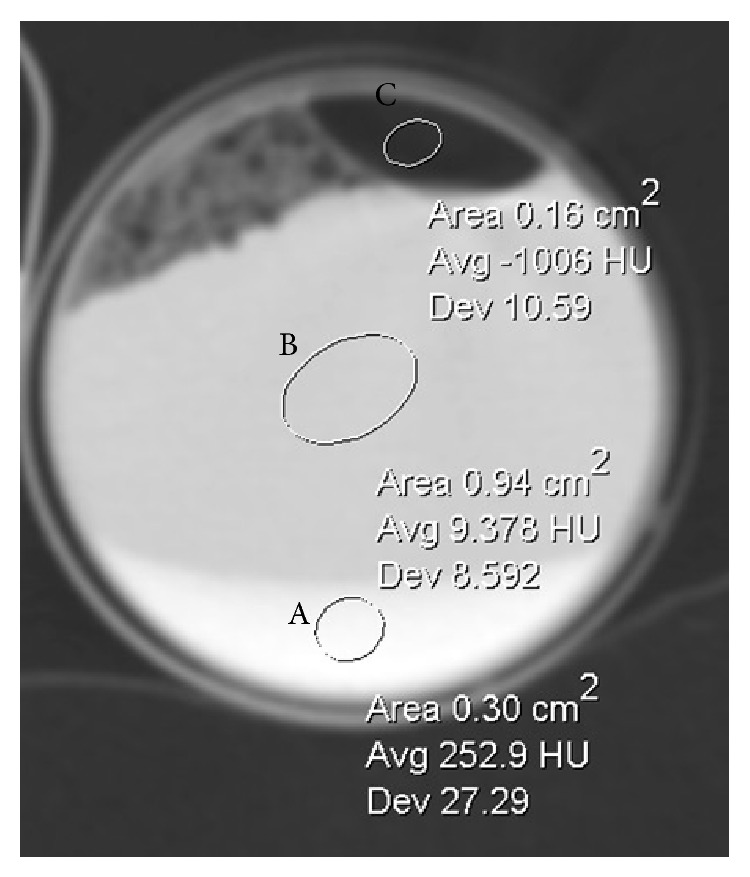
High-attenuation of talc. CT image of a syringe of saline mixed with talc demonstrates high density sediment of the talc (A) and fluid density of the supernatant saline (B). Note also the gas at the top (C).

The CT attenuation of the intrathoracic lymph nodes was not significantly different when compared to attenuation for areas of pleural thickening, suggesting that these are manifestations of a common process. It is widely believed that the high-attenuation is secondary to granulomatous inflammation incited by talc exposure; however, in our study, both high-attenuation pleural thickening and lymph nodes were observed as early as a single day after talc pleurodesis in one patient and within 10 days after talc pleurodesis in 6 patients. Size of high-attenuation lymph nodes and pleural thickening also persisted up to 38 months after talc pleurodesis in instances where followup chest CTs and PET/CTs were available. The presence of high-attenuation immediately following talc pleurodesis and stability of attenuation thereafter, suggests that deposition of talc, which is of high-attenuation (Figure 3), as well as granulomatous inflammation, may contribute to the characteristic appearance of high-attenuation lymph nodes and pleural thickening. The observed FDG avidity, however, is likely related to inflammatory changes. 

It is unclear why only 11 of 44 (25%) patients showed high-attenuation nodes, even when all demonstrated high-attenuation pleural thickening. The threshold of 100 HU we established for presuming talc deposition reflected the intrinsic high-attenuation of talc (Figure 3) and published data suggesting that benign nodes are typically higher (on average 75 HU) attenuation than metastatic nodes [8]. It is likely that if the attenuation threshold was lower, we could have detected a higher rate of involvement, although the specificity for talc involvement and confidence for excluding metastases would similarly be lower. It is also possible that high-attenuation nodes are only shown in some but not all patients with history of talc pleurodesis because of variable degree of talc dissemination in each patient, but this hypothesis would require additional studies to validate. 

One important limitation to our study was that it was retrospective in design, and so we do not have pathologic correlation for areas of high-attenuation pleural thickening or lymph nodes. The majority of our patients had known lung cancer that was advanced stage III or stage IV disease at diagnosis (75%), obviating the need for additional tissue sampling. We only studied lymph nodes and areas of pleural thickening that were of high-attenuation. In rare instances, calcified lymph nodes can represent metastases from mucinous ovarian carcinoma, colon carcinoma, bronchogenic carcinoma, papillary thyroid, or osteosarcoma [9]. However, on serial PET/CTs or chest CTs alone, which were available for 37 patients (84%), including all patients with high-attenuation lymph nodes, both size and attenuation of high-attenuation lymph nodes or pleural thickening did not change significantly over time, decreasing the likelihood that these represented metastatic deposits. We also measured attenuation values on noncontrast CTs, thereby eliminating the possibility of misinterpreting enhancing nodes as high-attenuation nodes. 

Because all of the lymph nodes were subcentimeter, drawing ROIs and obtaining accurate attenuation values or SUVs was challenging, particularly for anterior peridiaphragmatic and peri-IVC lymph nodes, which are in regions susceptible to misregistration and volume and count averaging related to respiration. Fortunately, the attenuation and SUV values we obtained in these regions were not significantly different compared to other lymph nodal regions, far from the diaphragm. 

## 5. Conclusion 

Our study adds to existing knowledge regarding potential pitfalls in the interpretation of FDG PET/CT scans in the thorax after talc pleurodesis. FDG avidity to high-attenuation lymph nodes following talc pleurodesis is not an uncommon observation and should not be mistaken for nodal metastases in patients with a history of lung cancer. 

## Figures and Tables

**Table 1:** Patient demographics, stratified by all patients, patients with FDG avid high-attenuation lymph nodes (avid high att. LNs), and patients who do not.

	Total patients		WITH avid high att. LNs		Without avid high att. LNs	
Number	44	100%	11	25%	33	75%
Age (years)						
Mean	64		59		67	
Stdev	12		11		13	
Range	34–81		45–74		34–81	
Sex						
Male	21	47%	6	55%	15	45%
Female	23	53%	5	45%	18	55%
Indication for talc pleurodesis						
Malignant pleural effusion	17	39%	4	36%	13	39%
Nonmalignant indication	27	61%	7	64%	20	61%
Indication for PET/CT						
Malignancy surveillance	41	93%	11	100%	30	91%
Sarcoidosis	1	2%	0	0%	1	3%
Search for occult malignancy	2	4%	0	0%	2	6%
Histology						
NSCLC	40	98%	11	100%	29	97%
SCLC	1	2%	0	0%	1	3%
Stage at initial diagnosis						
Stage I	4	10%	2	18%	2	6%
Stage II	4	10%	4	36%	0	0%
Stage III	11	25%	2	18%	9	27%
Stage IV	22	50%	3	28%	19	0.58
Interval from pleurodesis to PET/CT (months)						
Mean	28		34		26	
Stdev	37		31		38	
Range	0–117		0–58		0–117	
Clinical followup (months)						
Mean	15		12		16	
Stdev	11		8		11	
Range	0–52		6–38		0–52	

**Table 2:** Size, attenuation, and uptake of high-attenuation lymph nodes, stratified by nodal station.

	Overall	Peridiaphragmatic	Paracardiac	Internal mammary	Peri-IVC
Number	**22**	**11**	**5**	**4**	**2**
Size (mm)	5.1 ± 1.6	5.2 ± 1.3	6.2 ± 1.6	3.3 ± 1	5.5 ± 2.1
Attenuation (HU)	130 ± 29	131 ± 34	120 ± 29	129 ± 25	148 ± 11
SUV	2.6 ± 1.2	2.3 ± 1.1	2.4 ± 1	2.6 ± 1	2 ± 0.2
% “hot” by visual inspection	100%	100%	100%	100%	100%
